# Efforts to Identify and Combat Antimicrobial Resistance in Uganda: A Systematic Review

**DOI:** 10.3390/tropicalmed6020086

**Published:** 2021-05-24

**Authors:** Mark Tefero Kivumbi, Claire J. Standley

**Affiliations:** 1 School of Biosecurity, Biotechnical and Laboratory Sciences, Makerere University, Kampala P.O. Box 7062, Uganda; mark.kivumbi@students.mak.ac.ug; 2 Center for Global Health Science and Security, Georgetown University, Washington, DC 20057, USA

**Keywords:** antimicrobial resistance, antimicrobial stewardship, antiviral resistance, antibacterial resistance, antimalarial resistance, antifungal resistance, One Health, Uganda

## Abstract

The global burden of antimicrobial resistance is on the rise, resulting in higher morbidity and mortality in our communities. The spread of antimicrobial resistance in the environment and development of resistant microbes is a challenge to the control of antimicrobial resistance. Approaches, such as antimicrobial stewardship programmes and enhanced surveillance, have been devised to curb its spread. However, particularly in lower- and middle-income countries, the overall extent of antimicrobial resistance and knowledge on ongoing surveillance, stewardship or investigation efforts, are often poorly understood. This study aimed to look at the efforts that have been undertaken to detect and combat antimicrobial resistance in Uganda as a means of establishing an overview of the situation, to help inform future decisions. We conducted a systematic literature review of the PubMed database to assess these efforts. A search combining keywords associated with antimicrobial resistance were used to find relevant studies between 1995 and 2020 on surveillance of antimicrobial resistance in Uganda, and susceptibility of microbes to different drugs. The search yielded 430 records, 163 of which met the inclusion criteria for analysis. The studies were categorized according to country and region, the type of antimicrobial resistance, context of the study, study design and outcome of the study. We observed that antibacterial resistance and antimalarial resistance had the most published studies while antiviral and antifungal resistance were represented by very few studies each. Most studies were conducted in humans and hospital settings, with few in veterinary and One Health contexts, and only one that included environmental sampling. The majority of studies have focused on surveillance, susceptibility testing or resistance genes; none of our included papers had a policy or stewardship focus. The results from our work can inform public health policy on antimicrobial stewardship as it contributes to understanding the status of antimicrobial resistance surveillance in Uganda, and can also help to guide future research efforts. Notably, a One Health approach needs to be followed with respect to surveillance of antimicrobial resistance to better understand the mechanisms of resistance transfer across the human-animal–environment interface, including additional investigation in antiviral and antifungal resistance.

## 1. Introduction

Antimicrobial resistance (AMR) is a phenomenon where bacteria, fungi, parasites and viruses that previously were responsive to medicines evolve to become less or unresponsive to these treatments, increasing the risk of disease spread, treatment failure, severe illness and sometimes death [1,2]. The rapid evolution and spread of drug resistant microbes that acquire novel resistance mechanisms is a regular threat to our ability of treating simple infections like urinary tract infections and also more severe infections like bacteremia, tuberculosis and pneumonia that are life threatening [3,4]. There is also a rapid global spread of multi and pan-resistant microbes that are not responsive to most if not all available treatments [5]. Moreover, AMR can have a substantial economic burden, and also significantly affect national health systems, due to its effect on productivity of patients and/or their caretakers through prolonged stay in hospitals and the need for more expensive drugs as well as the need for intensive care treatment. Redundancy in prevention and inadequate treatment strategies against superbugs, and insufficient access to existing and new antimicrobials can result in high rates of treatment failure and even death in some scenarios, which will disproportionately impact those countries with more limited resources. Delicate medical procedures like surgery, cancer therapy, organ transplants and others, will become increasingly riskier and may result in death.

AMR is accelerated by clinical, biological, social, political, economic and environmental factors affecting both man, animals and the ecosystem [6]. The main drivers of AMR in developing countries, some of which also act as drivers in higher-income contexts, range from misuse and overuse of antimicrobials, self-medication, over prescription of antibiotics, high infection rates, use of antibiotics in livestock and fish farming, inadequate access to clean water facilities, sanitation and hygiene for man and animals, poor infection prevention and control strategies in the community, inadequate access to medical supplies like diagnostics, vaccines and effective drugs, ignorance, lack of medicine regulatory policies and poor enforcement of health regulation policies by relevant authorities [7,8,9,10], hunger and malnutrition, civil conflicts and poverty [7]. As drivers for AMR span both human and animal health, with strong environmental components as well, it is increasingly being viewed as a “One Health” issue, requiring multisectoral collaboration to establish effective surveillance and stewardship initiatives [11].

Uganda is a low-income country [12] situated in East Africa, and a member of the East African Community. Agriculture is a mainstay of the economy, with over 80% of the population estimated to engage in agricultural activities, although relatively little is intensive production. As a result of substantial health sector reforms initiated in the 1980s, more Ugandans now have access to basic healthcare services, including essential medicines, than ever before, although issues of quality and out of pocket expenses remain [13]. Antibiotics are widely available in local pharmacies, with rising concerns related to informal and unprescribed usage [14]. Over recent years, these factors have been suspected to be leading towards a growing trend of AMR and a decrease in positive treatment outcomes, with use of available medicines for both man and animal in Uganda [15]. In 2017, a World Health Organization-led Joint External Evaluation revealed weaknesses in Uganda’s efforts to address antimicrobial surveillance, highlighting that while detection of priority pathogens occurs, there is little coordination between sectors or operational guidance to support the country’s National Antimicrobial Resistance Action Plan [16], and also noted an absence of data on AMR activities within the veterinary sector [17]. The objective of this study was to determine the extent to which studies investigated AMR in Uganda, and to elicit information about trends in how these studies are undertaken that might help inform efforts to combat antimicrobial resistance in the country, including multisectoral coordination efforts, antimicrobial stewardship, policies and surveillance of resistance.

## 2. Materials and Methods

### 2.1. Systematic Literature Search

We carried out a systematic search in the PubMed database for publications on antimicrobial resistance, stewardship and antimicrobials in Uganda. The search query contained synonyms that included “Stewardship”, “Resistance”, “Resistant”, “Antimicrobial”, “antimicrobials”, “antibacterial”, “antibacterials”, “antibiotic”, “antibiotics”, “antivirals”, “antiviral”, “antimalarial”, “antimalarials”, which were combined with Uganda to be able to return relevant studies. The references in the publications were also reviewed to see if they were relevant to the study, per the same criteria, and subsequent snowball searches performed. The complete search syntax is available in a supplementary file (Supplementary Table S1). Our last search was carried out towards the end of August 2020. We did not put any restrictions on language and affiliate institutions or multicountry studies to minimize bias.

### 2.2. Selection of Papers

Articles included in the research had to meet four predetermined criteria, notably: (i) discussion of antimicrobial stewardship and/or antimicrobial resistance surveillance, and/or antimicrobial agents including but not limited to: objectives of the study, the title of the study or appearance in the abstract of the study paper; (ii) study location in Uganda, or multi-country studies with sample and/or data collection sites in Uganda; (iii) published after 1994 (before 1995, Uganda was faced with political unrest and instability, which likely provided a less than conducive environment for research; indeed, health research increased dramatically in subsequent years); and (iv) the outcome was on or included at minimum one of the following topics: resistance genes, antimicrobial resistance surveillance, policy-making or susceptibility testing. Both authors independently screened titles and article abstracts. One author screened full texts. Discrepant articles were thoroughly screened, reviewed and discussed between the two authors until a unanimous decision on their inclusion was reached.

### 2.3. Data Analysis

We characterized the included articles based on Uganda as a geographic location (including the district, where this information was available; if the study included other countries it was classified as “multicountry”), the type of antimicrobial resistance described (antibacterial, antiviral, antifungal or antimalarial), context of the study (human, veterinary or One Health, meaning considering both human and animal populations), study design (field or laboratory study; note “field” study includes hospital and clinical settings) and outcome of the study (focus on resistance genes, antimicrobial resistance surveillance, policy and/or susceptibility testing). We did not carry out meta-analysis of the data due to the diversity of the study types and identified data, and therefore instead present descriptive findings below.

## 3. Results

**Figure 1:**
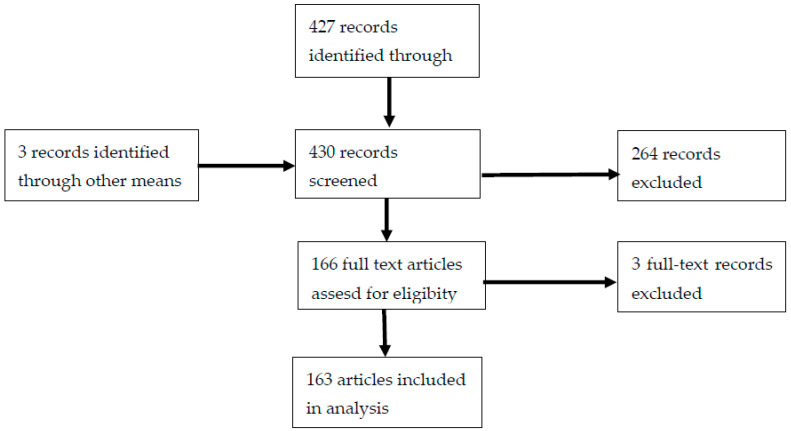
Preferred Reporting Items for Systematic Reviews and Meta-Analyses (PRISMA) study flowchart for our identified articles.

We identified a total of 427 titles published from 1995 to 2020 from the PubMed search. An additional three studies were identified through reference screening and snowball searches leading to a total of 430 records. They were screened for inclusion criteria with duplicates removed, leaving a total of 166 articles for full text screening. Of these, three were excluded for failing to meet the inclusion criteria, leaving a total of 163 articles included in our final analysis (Figure 1). We observed that research related to AMR surveillance was often performed interchangeably with susceptibility studies, and thus combined these topics for the purpose of reporting the findings. Table 1 summarizes the characteristics of the analyzed articles; a full list of the included articles and categorization is provided in Supplementary Table S2.

### Study Characteristics

**Figure 2:**
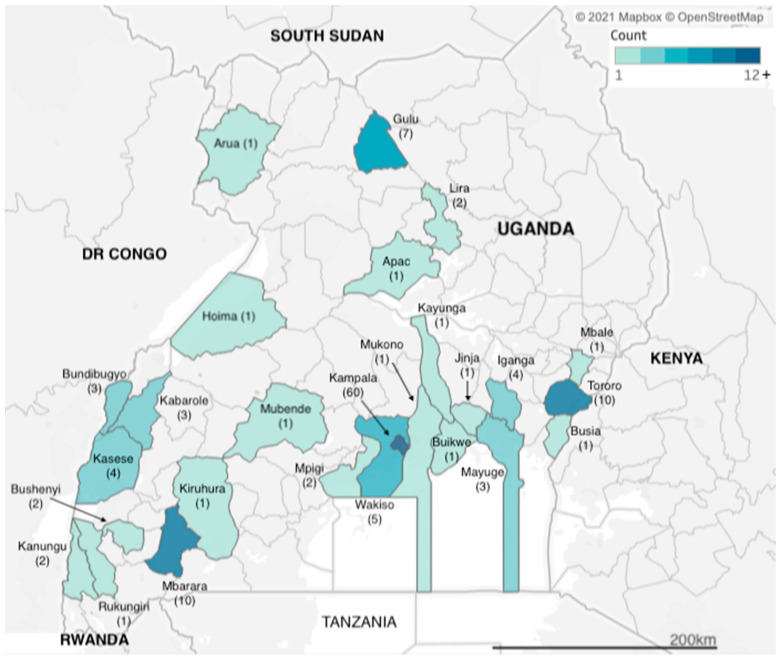
Geographic distribution of included studies.

We identified articles related to AMR from research carried out across two dozen districts in Uganda (Figure 2). However, more than a third of the studies (*n* = 60) were carried out in Kampala, which is also the capital city and where the national referral hospital, Mulago Hospital, is located. The next three most frequently observed study sites were Tororo, Mbarara and Gulu districts (*n* = 10, *n* = 10 and *n* = 7 studies), each of which has a regional referral hospital, for western, eastern and northern Uganda respectively. Additional districts with multiple identified studies included Kasese in western Uganda and Iganga in eastern Uganda (both *n* = 4 studies), both of which have large district hospitals that serve large communities; Bundibugyo and Kabarole districts (both *n* = 3 studies), which are situated in the western cattle corridor bordering the Democratic Republic of Congo. Studies carried out in Uganda totalled 155, while eight of the studies were multicountry studies, with Mayuge district (*n* = 3 studies) in eastern Uganda.

**Figure 3:**
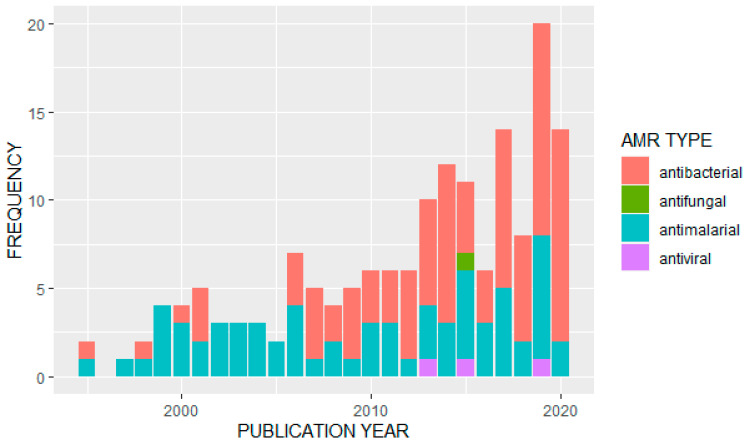
Distribution of studies on different AMR types by publication year.

We observed an overall increase in the frequency of published studies on AMR in Uganda over time (Figure 3). Of the 163 articles analyzed, the majority (*n* = 91) reported data on antibacterials or antibiotics. Of these, the most frequently studied bacterial pathogens were *Escherichia coli* (*n* = 13) and *Staphylococcus aureus* (*n* = 11). A further eight studies looked at *Salmonella* species, *Streptococcus* pneumonia was also covered in eight studies, and seven studies were on tuberculosis (*Mycobacterium tuberculosis*). *Klebsiella pneumoniae* had four studies while *Enterococci* species, *H. influenza*, *V. cholera* (cholera) and *H. pylori* were also covered by identified papers. Major antibiotics that were used for surveillance of antimicrobial resistance included: penicillin, tetracycline, ampicillin, chloramphenicol, ciprofloxacin, trimethoprim, sulfonamide, ceftriaxone, gentamicin, vancomycin, erythromycin, oxacillin, methicillin, clarithromycin, sulfamethoxazole/trimethoprim and other fluoroquinolones.

Of the other types of antimicrobials covered in the identified studies, 68 papers reported data on antimalarials. Twenty-one studies reported primarily on resistance to artemisinin derivatives and/or common components in artemisinin-based combination therapies; 18 studies reported on resistance to multiple drugs or drug classes, with common combinations including chloroquine and sulfadoxine/pyrimethamine or looking for multiple resistance genotypes. Fifteen studies focused primarily on resistance to sulfadoxine/pyrimethamine or other antifolates or sulfonamides, and thirteen studies focused on genotypes or phenotypes associated specifically with chloroquine resistance. One study did not look at a specific antimalarial treatment, but associated strain diversity with treatment failure. Only three studies were identified that reported on antiviral resistance, of which two focused on the hepatitis virus (one on hepatitis C, and the other in hepatitis B in patients co-infected with HIV and undergoing antiretroviral therapy), and one focused on resistance to antiretroviral treatment. We located only a single study that discussed antifungals, on *Cryptococcus neoformans*.

There was similarly a heavy emphasis on the human health sector in the studies we identified, with 145 studies (over 88%) reporting data from human subjects, 12 studies focused on animal subjects (five focused on cattle, five on chickens’ and two on pigs/swine), while only six identified studies used a One Health approach. Of these, four studies looked at animal workers and their animals (three of which were cattle, and one was chickens), and two looked at animals and humans in the same geographic locations; the first covered a broad variety of animal species (cattle, goats, pigs, sheep and non-human primates), while the second focused more narrowly on pigs and birds, but also included environmental sampling from ponds, animal waste and sewage. This was the only study we identified that included environmental surveillance. All the veterinary and One Health studies focused on bacterial pathogens and/or antibiotic resistance, with the most commonly targeted pathogens being *E. coli* and *Salmonella* spp., with five and six studies, respectively, focusing exclusively on that pathogen. One study looked both at *E. coli and Salmonella* (a study in dairy cattle), and five studies looked more broadly across different types of bacterial pathogens or antibiotic resistance genes and phenotypes. Overall, there was a strong food safety focus, with all but one of the veterinary and One Health studies focused on livestock and food-producing animals; only one study also considered wildlife (non-human primates in national parks adjacent to agricultural areas and human habitation). We observed that the veterinary and One Health studies were all relatively recently published, with the earliest dating from 2013, and the majority published in 2019 and 2020.

Overall, only ten of the studies were laboratory-based, whereas the remaining 153 studies were done in the field, with recruitment specifically taking place in clinical/hospital settings. None of the human-focused studies appeared to include surveillance or recruitment at the community level, although all the veterinary and One Health studies were conducted in communities. Eighty-three of the studies produced results on resistance genotypes (Table 2), while 80 studies focused on simply looking at resistance profiles of the microbes, for example, methicillin-resistant *Staphylococcus*, riphampicin-resistant tuberculosis, vancomycin-resistant *Staphylococcus aureus*, beta lactamase-resistant *E. coli* and carbapenem-resistant *Klebsiella pneumoniae*. None of the articles we identified described the process or outcome of AMR stewardship initiatives, or focused on policy aspects of AMR prevention, mitigation or management, beyond noting policy changes with respect to antimalarial use, for example, as a motivation for continuing to surveil for resistance phenotypes and genotypes.

## 4. Discussion

Our systematic literature revealed that substantial work has been done in Uganda to investigate the emergence and spread of AMR over the 25 years. However, we observed large differences in terms of the type of AMR investigated, the settings in which those studies were conducted and the locations of the studies, with the majority of studies performed in urban areas and large-scale health facilities. We observed very few studies that had been performed in rural settings, and many districts were not covered at all in the available literature. We also identified a handful of articles in which Uganda was included as part of a multicountry study, but in these cases, the specific district or location of the study or sample collection was rarely mentioned, limiting the application of the study’s findings for the specific context within Uganda. The high frequency of studies in Uganda is in contrast to many other countries in Africa; a 2017 systematic review reported that about 42% of African countries do not have published studies on AMR [18].

The review further demonstrated that a great number of infectious diseases have been shown to resist available and routine therapy in the Ugandan context, for example, tuberculosis [19,20,21], pneumonia [22,23], salmonellosis [24,25], malaria [26,27], gonorrhea [28,29,30] and other urinary tract infections and respiratory infections, as well as important viral and fungal infections. There has been detection of wide spread beta lactam and non-beta lactam antibiotic resistance reported in district referral hospitals [31] as well as multidrug resistance amongst clinical isolates [32,33]. Carbapenem resistance has also emerged and has been detected for *K. pneumonia and E. coli*. as well as other pathogens, together with methicillin-resistant *S. aureus* and extended-spectrum beta-lactamase producing bacteria. Collectively, these findings demonstrate the substantial threat posed, particularly by antibiotic resistance, in Uganda, with bacteria demonstrating resistance to even new and extended spectrum or novel, and more efficacious, antibiotics [34].

Most of the studies from other countries in sub-Saharan Africa focused on antibacterial resistance, with few or no studies in areas of antivirals and antifungals, and moderate numbers of studies on antimalarials, which aligns with our observations from Uganda [18,35]. Countries with a high prevalence of HIV tended to have higher numbers of studies on HIV viral resistance [36]; Uganda’s HIV prevalence was estimated in 2017 at just over 6%, with higher levels in urban versus rural areas, and women disproportionately impacted [37]. While not as high as HIV prevalence levels in other countries in Africa, this still represents a substantial burden of disease, and it is was surprising therefore to identify just a single study focused on antiretroviral resistance in Uganda [38], particularly given concerns around emerging and spreading resistance to antiretroviral therapies in the African region [39]. We similarly identified only one article examining antifungal resistance in Uganda [40]. Globally, an estimated 1.7 million die each year from fungal infections [41], which is a similar number as for tuberculosis and malaria combined, and yet antifungal resistance has only recently been integrated into global AMR initiatives. Antifungals are similarly not mentioned in Uganda’s AMR National Action Plan [16].

We also observed that most studies on AMR in Uganda focused on resistance in human beings, neglecting animals and the environment, despite the known importance that all three sectors play in preventing and mitigating the spread of AMR. However, there appeared to be increasing interest in the topic of animal and environmental studies on AMR, perhaps even stemming from increased attention paid to AMR issues after the launch of the Global Health Security Agenda in 2014, of which Uganda is a member country. In Uganda, the establishment of the National One Health Platform in November 2016 presaged a strong emphasis on multisectoral coordination across a number of areas of infectious disease control, and with a particular emphasis on emerging zoonotic diseases [42]. While AMR is highlighted as a key priority area of the National One Health Platform, which moreover is the designated responsible body for implementation of the AMR National Action Plan, the continuing lack of published One Health studies on AMR in Uganda suggests additional efforts will be required to encourage greater integration and coordination in this area. Indeed, while One Health is mentioned as an approach within the AMR National Action Plan, it is not listed directly as an objective in its own right. As noted previously, Uganda’s Joint External Evaluation in 2017 mentioned the absence of available data on AMR in the environmental and veterinary sectors as a limitation for providing specific recommendations to strengthen AMR control and the need for greater cross-sectoral coordination [17]; these deficits must be addressed. The strong existing emphasis on food safety aspects of AMR surveillance among the identified veterinary and One Health studies further suggests opportunities for synergies between the Food Safety, Zoonotic Disease and AMR technical areas under the JEE, which should be explored further from an implementation research and capacity strengthening perspective.

In Uganda, the majority of the studies we identified focused on specimens collected from patients at different clinical health facilities, and especially in larger district or regional hospitals, while a much smaller number used archived laboratory specimens. Similarly, analysis of the studies revealed that the outputs largely focused on resistance profiles or detection of resistance genes. While this information is very important in the surveillance of AMR, particularly for guiding decisions on appropriate treatment protocols for patients, without analyses of the broader drivers of AMR, or studies conducted outside of acute clinical settings, the results may have limited broader applicability for the development of policies and practices to mitigate further AMR emergence. We noted few studies that integrated multiple forms of data or attempted mixed methods approaches for understanding drivers of AMR, or that focused on the implications or impacts of policy initiatives related to AMR. However, this may be changing, with studies planned that will explicitly look across social, biological and community-level drivers of AMR in Uganda and neighbouring countries [43].

Based on our findings, we recommend that efforts be taken to conduct surveillance for AMR more broadly across different regions in Uganda, and across a more diverse array of rural and urban settings, to include community cross-sectional studies, and incorporate investigations into attitudes and practices as well as resistance profiles and genetics. We encourage that more attention be paid to antiviral and antifungal resistance; a key step in this regard would be to include antifungal resistance as a topic area under the AMR National Action Plan and related policy documents. Similarly, where possible, researchers should be encouraged to collaborate across sectors to approach AMR from a One Health perspective and move beyond only considering AMR in a food safety context, although this may prove a fruitful area for cross-sectoral collaborative research as well. The National One Health Platform should encourage researchers to consider One Health methodologies that explicitly look at outcomes across human, animal (wildlife as well as domestic species) and environmental health indictors; frameworks such as the Checklist for One Health Epidemiological Reporting of Evidence can be particularly helpful in assisting researchers in guiding the design, analysis and dissemination of multisectoral studies, for AMR as well as other One Health issues [44]. Finally, the wealth of research on AMR in Uganda must not be sequestered in the scientific literature, but rather utilized to inform policy and practice. To this end, initiatives such as the National Antimicrobial Resistance Conferences, each hosted by different Ugandan universities or public health-affiliated agencies, and supported by the Africa One Health University Network (formerly the One Health Central and East Africa University Network), present an ideal mechanism for broader sharing of knowledge and novel research related to AMR, particularly in a One Health context, provided policy- and decision-makers are included as participants. In this way, research data, across human, animal and environmental contexts, can be more directly utilized for the development and implementation of policies and laws to control the spread of AMR in Uganda and in neighbouring countries.

As in all research, our study was subject to certain limitations. Our inclusion date of papers from 1995 onwards was based on our judgment of the research environment during Uganda’s prior years of political instability, and the likelihood of older studies having limited relevance in the modern context, but it is possible that earlier papers could still shed light on patterns of AMR in Uganda. Similarly, our search focused on peer-reviewed publications, and had limited scope for inclusion of “grey” literature or governmental and non-governmental reports. These non-peer reviewed publications may be particularly important for understanding policy and stewardship initiatives, although we suggest there should still be efforts to ensure that the outcomes of policy and control efforts are also captured in academic literature, to facilitate sharing of lessons learned, best practices and allow for greater translation of successful models to new contexts.

## Figures and Tables

**Table 1:** Summary of characteristics identified across included papers.

Resistance Type	Number of Articles	AMR Context			Study Design		Output		
		Human	Veterinary/Animal	One Health	Field Study	Lab Study	Surveillance or Susceptibility	Resistance Genes	Policy
Antibacterial	91	73	12	6	82	9	66	25	0
Antimalarial	68	68	0	0	67	1	13	55	0
Antiviral	3	3	0	0	3	0	0	3	0
Antifungal	1	1	0	0	1	0	1	0	0
Total	163	145	12	6	153	10	80	83	0

**Table 2:** Resistance Genes Identified in the Included Studies.

Resistance Type	Target Pathogen	Number of Studies	Examples of Resistance Genes Identified
Antibacterial	*E. coli*	7	blaCTX-M, blaACT, arnA, integrons class 1 and 2, qnrS1, tetA, tetB, sul2, blaSHV, blaTEM
	*Staphylococcus* spp.	8	spa types t064, t037, SCCmec types I and IV, mecA, aac(6′)-Ie-aph(2′’)-Ia, aph(3′)-IIIa, ant(4′)-Ia, blaZ, mecA, vanA, vanB1
	*Streptococcus* spp.	3	dihydropteroate synthase (DHPS) and dihydrofolate reductase (DHFR), folA and folP genes,
	*Salmonella* spp.	2	blaTEM-1,cmlA, tetA, qnrS, sul1, dhfrI, dhfrVII
	*Mycobacterium* spp.	5	Mutation gyrA Genotype Uganda I and II has Thr80Ala (acc/gcc), rpoB gene mutations
	*Klebsiella* spp.	1	blaCTX-M, blaSHV, blaTEM
	*Enterococcus* spp.	1	EBC, FOX, ACC, CIT, DHA, MOX
Antimalarial	*Plasmodium* *falciparum*	55	Pfmdr1 N86Y, Y184F and D1246Y, Pfpm2, PfKelch13, plasmepsin2 gene, pfcrt 76T, Pfdhfr, Pfdhps
Antiviral	Hepatitis C virus	1	g4 and g7 strains contain nonstructural (ns) protein 3 and 5A polymorphisms associated with resistance to DAAs
	Hepatitis B virus	1	rtM204V/I mutations
	HIV	1	Thymidine analog mutations, M184V

## Data Availability

All data described are available in the manuscript or Supplemental Material. Full details on the excluded articles are available upon reasonable request to the corresponding author.

## Supplementary Materials

The following are available online at https://www.mdpi.com/article/10.3390/tropicalmed6020086/s1, Table S1: Full search syntax for the systematic review, Table S2: Table of 163 included articles and analysis findings.

## References

[1] Clarke C.R. (2006). Antimicrobial Resistance. Vet. Clin. N. Am. Small Anim. Pract..

[2] Morrison L., Zembower T.R. (2020). Antimicrobial Resistance. Gastrointest. Endosc. Clin. N. Am..

[3] Boucher H.W., Talbot G.H., Bradley J.S., Edwards J.E., Gilbert D., Rice L.B., Scheld M., Spellberg B., Bartlett J. (2009). Bad Bugs, No Drugs: No ESKAPE! An Update from the Infectious Diseases Society of America. Clin. Infect. Dis..

[4] Kuehn B.M. (2013). “Nightmare” Bacteria on the Rise in US Hospitals, Long-Term Care Facilities. JAMA J. Am. Med. Assoc..

[5] Walsh T.R., Toleman M.A. (2012). The Emergence of Pan-Resistant Gram-Negative Pathogens Merits a Rapid Global Political Response. J. Antimicrob. Chemother..

[6] Vikesland P., Garner E., Gupta S., Kang S., Maile-Moskowitz A., Zhu N. (2019). Differential Drivers of Antimicrobial Resistance across the World. Acc. Chem. Res..

[7] Byarugaba D.K. (2004). Antimicrobial Resistance in Developing Countries and Responsible Risk Factors. Int. J. Antimicrob. Agents.

[8] UNAS (2015). Antibiotic Resistance in Uganda: Situation Anaysis.

[9] Odoi R., Joakim M., Resistance A. (2019). Anti-Microbial Resistance in Uganda. AMR.

[10] WHO | Antimicrobial Resistance. https://www.who.int/antimicrobial-resistance/en/.

[11] McEwen S.A., Collignon P.J. (2018). Antimicrobial Resistance: A One Health Perspective. Antimicrobial Resistance in Bacteria from Livestock and Companion Animals.

[12] Uganda | Data. https://data.worldbank.org/country/UG.

[13] Okech T.C. (2014). Analytical Review of Health Care Reforms in Uganda and Its Implication on Health Equity. World J. Med. Med. Sci. Res..

[14] Mbonye A.K., Buregyeya E., Rutebemberwa E., Clarke S.E., Lal S., Hansen K.S., Magnussen P., LaRussa P. (2016). Prescription for Antibiotics at Drug Shops and Strategies to Improve Quality of Care and Patient Safety: A Cross-Sectional Survey in the Private Sector in Uganda. BMJ Open.

[15] Ikwap K., Erume J., Owiny D.O., Nasinyama G.W., Melin L., Bengtsson B., Lundeheim N., Fellström C., Jacobson M. (2014). Salmonella Species in Piglets and Weaners from Uganda: Prevalence, Antimicrobial Resistance and Herd-Level Risk Factors. Prev. Vet. Med..

[16] (2016). Uganda National Academy of Sciences (UNAS) Antimicrobial Resistance National Action Plans. Gov. Uganda.

[17] World Health Organization Joint External Evaluation of IHR Core Capacities of the Republic of Uganda: Mission Report: June 26–30.

[18] Tadesse B.T., Ashley E.A., Ongarello S., Havumaki J., Wijegoonewardena M., González I.J., Dittrich S. (2017). Antimicrobial Resistance in Africa: A Systematic Review. BMC Infect. Dis..

[19] Izudi J., Tamwesigire I.K., Bajunirwe F. (2020). Surveillance for Multi-Drug and Rifampicin Resistant Tuberculosis and Treatment Outcomes among Previously Treated Persons with Tuberculosis in the Era of GeneXpert in Rural Eastern Uganda. J. Clin. Tuberc. Other Mycobact. Dis..

[20] Kigozi E., Kasule G.W., Musisi K., Lukoye D., Kyobe S., Katabazi F.A., Wampande E.M., Joloba M.L., Kateete D.P. (2018). Prevalence and Patterns of Rifampicin and Isoniazid Resistance Conferring Mutations in Mycobacterium Tuberculosis Isolates from Uganda. PLoS ONE.

[21] Naluyange R., Mboowa G., Komakech K., Semugenze D., Kateete D.P., Ssengooba W. (2020). High Prevalence of Phenotypic Pyrazinamide Resistance and Its Association with PncA Gene Mutations in Mycobacterium Tuberculosis Isolates from Uganda. PLoS ONE.

[22] Taylor S.M., Meshnick S.R., Worodria W., Andama A., Cattamanchi A., Davis J.L., Yoo S.D., Byanyima P., Kaswabuli S., Goodman C.D. (2012). Low Prevalence of Pneumocystis Pneumonia (PCP) but High Prevalence of Pneumocystis Dihydropteroate Synthase (Dhps) Gene Mutations in HIV-Infected Persons in Uganda. PLoS ONE.

[23] Nantanda R., Hildenwall H., Peterson S., Kaddu-Mulindwa D., Kalyesubula I., Tumwine J.K. (2008). Bacterial Aetiology and Outcome in Children with Severe Pneumonia in Uganda. Ann. Trop. Paediatr..

[24] Odoch T., Sekse C., L’abee-Lund T.M., Hansen H.C.H., Kankya C., Wasteson Y. (2018). Diversity and Antimicrobial Resistance Genotypes in Non-Typhoidal Salmonella Isolates from Poultry Farms in Uganda. Int. J. Environ. Res. Public Health.

[25] Afema J.A., Byarugaba D.K., Shah D.H., Atukwase E., Nambi M., Sischo W.M. (2016). Potential Sources and Transmission of Salmonella and Antimicrobial Resistance in Kampala, Uganda. PLoS ONE.

[26] Asua V., Conrad M.D., Aydemir O., Duvalsaint M., Legac J., Duarte E., Tumwebaze P., Chin D.M., Cooper R.A., Yeka A. (2020). Changing Prevalence of Potential Mediators of Aminoquinoline, Antifolate, and Artemisinin Resistance Across Uganda. J. Infect. Dis..

[27] Cuu G., Asua V., Tukwasibwe S., Nsobya S.L., Nanteza A., Kimuda M.P., Mpimbaza A., Rosenthal P.J. (2020). Associations between Aminoquinoline Resistance Genotypes and Clinical Presentations of Plasmodium Falciparum Infection in Uganda. Antimicrob. Agents Chemother..

[28] Florence P.A., Otim F., Okongo F., Ogwang M., Greco D. (2012). The Prevalence and Antibiotics Susceptibility Pattern of Neisseria Gonorrhoeae in Patients Attending OPD Clinics at St. Mary’s Hospital Lacor Uganda. J. Prev. Med. Hyg..

[29] Vandepitte J., Hughes P., Matovu G., Bukenya J., Grosskurth H., Lewis D.A. (2014). High Prevalence of Ciprofloxacin-Resistant Gonorrhea among Female Sex Workers in Kampala, Uganda (2008–2009). Sex. Transm. Dis..

[30] Workneh M., Hamill M.M., Kakooza F., Mande E., Wagner J., Mbabazi O., Mugasha R., Kajumbula H., Walwema R., Zenilman J. (2020). Antimicrobial Resistance of Neisseria Gonorrhoeae in a Newly Implemented Surveillance Program in Uganda: Surveillance Report. JMIR Public Health Surveill..

[31] Najjuka C.F. (2017). Characterization of Extended Spectrum Βlactamases Elaborated in Enterobacteriaceae in Uganda.

[32] Manson A.L., Cohen K.A., Abeel T., Desjardins C.A., Armstrong D.T., Barry C.E., Brand J., Chapman S.B., Cho S.N., Gabrielian A. (2017). Genomic Analysis of Globally Diverse Mycobacterium Tuberculosis Strains Provides Insights into the Emergence and Spread of Multidrug Resistance. Nat. Genet..

[33] Stanley I.J., Kajumbula H., Bazira J., Kansiime C., Rwego I.B., Asiimwe B.B. (2018). Multidrug Resistance among Escherichia Coli and Klebsiella Pneumoniae Carried in the Gut of Out-Patients from Pastoralist Communities of Kasese District, Uganda. PLoS ONE.

[34] Bebell L.M., Ayebare A., Boum Y., Siedner M.J., Bazira J., Schiff S.J., Metlay J.P., Bangsberg D.R., Ttendo S., Firth P.G. (2017). Prevalence and Correlates of MRSA and MSSA Nasal Carriage at a Ugandan Regional Referral Hospital. J. Antimicrob. Chemother..

[35] Ampaire L., Muhindo A., Orikiriza P., Mwanga-Amumpaire J., Bebell L., Boum Y. (2016). A Review of Antimicrobial Resistance in East Africa. Afr. J. Lab. Med..

[36] WHO (2016). HIV Drug Resistance Surveillance Guidance.

[37] Uganda AIDS Commission (2017). Uganda Population Based HIV Impact Assessment.

[38] Sigaloff K.C.E., Kayiwa J., Musiime V., Calis J.C.J., Kaudha E., Mukuye A., Matama C., Nankya I., Nakatudde L., Dekker J.T. (2013). Short Communication: High Rates of Thymidine Analogue Mutations and Dual-Class Resistance among HIV-Infected Ugandan Children Failing First-Line Antiretroviral Therapy. Aids Res. Hum. Retrovir..

[39] WHO Unveils Plan to Tackle Rising HIV Drug Resistance in Africa | WHO | Regional Office for Africa. https://www.afro.who.int/news/who-unveils-plan-tackle-rising-hiv-drug-resistance-africa.

[40] Smith K.D., Achan B., Hullsiek K.H., McDonald T.R., Okagaki L.H., Alhadab A.A., Akampurira A., Rhein J.R., Meya D.B., Boulware D.R. (2015). Increased Antifungal Drug Resistance in Clinical Isolates of Cryptococcus Neoformans in Uganda. Antimicrob. Agents Chemother..

[41] Kainz K., Bauer M.A., Madeo F., Carmona-Gutierrez D. (2020). Fungal Infections in Humans: The Silent Crisis. Microbial Cell.

[42] Buregyeya E., Atusingwize E., Nsamba P., Musoke D., Naigaga I., Kabasa J.D., Amuguni H., Bazeyo W. (2020). Operationalizing the One Health Approach in Uganda: Challenges and Opportunities. J. Epidemiol. Glob. Health.

[43] Asiimwe B.B., Kiiru J., Mshana S.E., Neema S., Keenan K., Kesby M., Mwanga J.R., Sloan D.J., Mmbaga B.T., Smith V.A. (2021). Protocol for an Interdisciplinary Cross-Sectional Study Investigating the Social, Biological and Community-Level Drivers of Antimicrobial Resistance (AMR): Holistic Approach to Unravel Antibacterial Resistance in East Africa (HATUA). BMJ Open.

[44] Davis M.F., Rankin S.C., Schurer J.M., Cole S., Conti L., Rabinowitz P., Gray G., Kahn L., Machalaba C., Mazet J. (2017). Checklist for One Health Epidemiological Reporting of Evidence (COHERE). One Health.

