# Supplementary material for: Efforts to Identify and Combat Antimicrobial Resistance in Uganda: A Systematic Review
Source: Trop Med Infect Dis. 2021 May 24;6(2):86. doi: 10.3390/tropicalmed6020086 (PMC8163190; doi:10.3390/tropicalmed6020086)
Supplement: Supplementary file 1 [file tropicalmed-06-00086-s001.zip › Supp1_Tefero_Search syntax_Uganda AMR.pdf]

Search syntax

("Stewardship" OR "resistance" OR "resistant") AND ("Antimicrobial" OR "antimicrobials" OR "antibacterial" OR "antibacterials" OR "antibiotic" OR "antibiotics" OR "antivirals" OR "antiviral" OR "antimalarial" OR "antimalarials") AND "Uganda"
